# Accumulation of NH_4_^+^ and NO_3_^−^ inside Biofilms of Natural Microbial Consortia: Implication on Nutrients Seasonal Dynamic in Aquatic Ecosystems

**DOI:** 10.1155/2019/6473690

**Published:** 2019-06-02

**Authors:** Andi Kurniawan, Tatsuya Yamamoto

**Affiliations:** ^1^Department of Aquatic Resources Management, University of Brawijaya, Malang 65145, Indonesia; ^2^Coastal and Marine Research Centre, University of Brawijaya, Malang 65145, Indonesia; ^3^College of Life Science, Ritsumeikan University, 1-1-1 Noji-Higashi, Kusatsu, Shiga, Japan

## Abstract

Microbial biofilms are ubiquitous in aquatic ecosystems. Inside the biofilm is the nutrient-rich microenvironment promoted by the accumulation of the nutrient ions such as NH_4_^+^ and NO_3_^−^ from surrounding water. The present study investigated the characteristics of NH_4_^+^ and NO_3_^−^ accumulation into the biofilm of natural microbial consortia collected from Lake Biwa, Japan. The results showed the following: (1) the concentrations of NH_4_^+^ and NO_3_^−^ inside the biofilm were much higher than those in the surrounding water; (2) the nutrient ion concentration inside the biofilm changed in synchrony with those in the surrounding water; (3) biofilm polymers have both positively and negatively charged sites; (4) electrostatic attractive interactions between the charged sites on biofilm polymers and oppositely charged ions outside the biofilm seem to play important roles in the accumulation of nutrient ions into the biofilm from the surrounding water; (5) the bacterial community structure differs between the biofilm and surrounding water. The present study revealed that the accumulation of nutrient ions into the biofilm indicates the removal of these ions from water outside the biofilm. According to the result of this study, accumulation of ions such as NH_4_^+^ and NO_3_^−^ into the biofilm of natural microbial consortia may have implications on nutrients seasonal dynamic in aquatic ecosystems.

## 1. Introduction

Biofilms are ubiquitous in aquatic environments and are formed when bacteria and other microorganisms attach onto a solid surface [1, 2]. Biofilms have been reported to have various important functions in the aquatic ecosystems such as in the purification of pollutants, as microbial gene pools, and in the nutrient cycling process [3]. One of the main processes that support these functions is the ion accumulation into the biofilm matrices.

Biofilms have been reported to have high sorption capacities for various ions [4, 5]. The ions that can be adsorbed into the biofilm include nutrient ions, such as NH_4_^+^ and NO_3_^−^ that are required by organisms in aquatic ecosystems including microbes inside the biofilms [6, 7]. However, the study that investigates the characteristics of microenvironment inside the biofilm of natural microbial consortia and its implication to the nutrient ions uptake process, as well as to the seasonal dynamic of the ions in the aquatic ecosystems, has rarely been conducted.

This study aims to characterize the microenvironment inside the biofilms formed in Lake Biwa, Japan (*i.e*., concentrations of NH_4_^+^ and NO_3_^−^, bacterial community structures, and electric charge properties), and the uptake process of NH_4_^+^ and NO_3_^−^ into the biofilms. The results indicate that the electrostatic interactions between the charged sites on biofilm polymers and oppositely charged nutrient ions outside the biofilm play essential roles in the accumulation of the nutrient ions inside the biofilms. Enrichment of the nutrient ions into the biofilm leads to the removal of these nutrient ions from the water outside the biofilm. The nutrient ions held inside the biofilm can be easily used by microbes and transformed into a biomass inside the biofilm resulting in the different bacterial community structure inside the biofilms compared to that of surrounding water [8, 9], and thus, the biofilm may continuously take up nutrient ions from surrounding water. According to the results of the present study, the accumulation nutrient ions such as NH_4_^+^ and NO_3_^−^ inside the biofilms of natural microbial consortia may have significant implications to the seasonal dynamics of the nutrient ions in the aquatic ecosystems.

## 2. Materials and Methods

### 2.1. Sampling Site and Sample Preparation

The samples in this study were biofilms formed on the surfaces of stones and reeds collected from the shore of the southern basin (Akanoiwan) of Lake Biwa, Japan. Several stones (granite, 10 cm × 10 cm × 10 cm; sterilized with 70% ethanol before setting) were placed adjacent to reeds (approximately 100 cm) more than 2 months before the sampling date to allow biofilm formation. Samples of the biofilms were collected to investigate the nutrient ion concentrations in March, June, September, and December of 2012. To investigate the bacterial community structures, the electric charge properties, and the nutrient enrichment mechanisms of the biofilm matrices, biofilm samples were collected from the surfaces of the stones or reeds in December 2012. Stones were taken from a depth of approximately 70 cm, and reeds were cut at a depth of approximately 10 cm from the water's surface. Both stones and reeds were carried to the laboratory in separate plastic containers filled with lake water collected nearby and maintained at 4°C. Water samples were also collected from areas close to the stones and reeds (approximately 50 cm).

The biofilms on the surfaces of the stones (approximately 3 stones in each sampling) and the reeds (approximately 10 pieces in each sampling) were removed using a sterilized toothbrush and suspended in sterilized distilled water. The biofilm pellets were prepared by centrifuging (8,000 × g at 4°C for 10 min) the biofilm suspensions, and the supernatants were used to measure the ion concentrations in the interstitial water of the biofilms.

### 2.2. DNA Extraction and Purification

The frozen biofilm suspension and lake water (1 mL) samples were placed in 1.5 mL Eppendorf tubes. The samples were dried in a desiccator under a vacuum for 12 h. The dried biofilm and the lake water residue were used for DNA extraction with QuickGene (QuickGene 800; Fujifilm, Tokyo, Japan) according to the manufacturer's instructions. A negative control without a sample was also prepared from the vacuuming step to check for contamination from the reagents and cross-contamination among the samples.

### 2.3. PCR-DGGE

Variable regions III and V of the 16S rDNA were amplified using the following primer set for bacteria: 341f-GC (*Escherichia coli* positions 341–357), 5′-CGCCCGCCGCGCCCCGCGCCCGTCCCGCCGCCCCCGCCCGCTACGGGAGGCAGCAG-3′ (the underlined sequence denotes the GC clamp) [10], and 907r (*Escherichia coli* positions 926–907), 5′-CCCCGTCAATTCATTTGAGTTT-3′ [11]. The PCR mixture contained 12.5 *μ*L of GoTaq (Promega, Madison, WI, USA), 2.0 *μ*L of each primer (10 pmol each), 3.5 *μ*L of Milli-QW, and 5 *μ*L of the DNA template in a total volume of 25 *μ*L. The PCR amplification was performed in a thermal cycler (iCycler; Bio-Rad Laboratories, Hercules, CA, USA). The amplification conditions were as follows: 95°C for 5 min, 80°C for 1 min (initial denaturing), 65°C for 1 min (annealing), 72°C for 1 min (extension), 30 cycles of 95°C for 1 min, 62°C for 1 min (with a decrease of 0.8°C at every cycle), and 72°C for 1 min, 9 cycles of 95°C for 1 min, 52°C for 1 min, and 72°C for 1 min, 94°C for 1 min, 55°C for 1 min, and a final extension step of 72°C for 10 min.

DGGE was performed in a 6% (w/v) acrylamide gel that contained a linear gradient of 30% to 60% denaturant (100% denaturant: 7 M urea and 40% (w/v) formamide). Aliquots (approximately 200 ng) of the PCR products were mixed with loading dye, loaded into the wells of the DGGE gel, and electrophoresed for 14 h at 100 V and 60°C using the DCode Universal Mutation Detection system (Bio-Rad Laboratories, Hercules, CA, USA). The DGGE marker (5 *μ*L, DGGE Marker II; Nippon Gene, Tokyo, Japan) was loaded onto both sides of the gel. After electrophoresis, the gel was soaked in SYBR Gold nucleic acid gel stain solution (Promega, Madison, WI, USA) for 30 min and photographed under UV transillumination using Printgraph (DT-20MP; ATTO, Tokyo, Japan). The experimental procedures from the DNA extraction to the analysis of the DGGE patterns were performed in duplicate using biofilm and lake water samples, and the DGGE patterns were confirmed to be identical in the duplicate samples. A cluster analysis of the DGGE band patterns was performed using band pattern analysis software (TotalLab, Shimadzu, Kyoto, Japan). The dendrogram was constructed using the unweighted pair-group method with the arithmetic mean (UPGMA).

### 2.4. Electrophoretic Mobility

One milliliter of the biofilm suspension (containing approximately 0.03 wet-g of biofilm) was placed in an electric field, and the electrophoretic mobility (EPM) of the dispersed biofilm was measured with a ZETASIZER Nano-Z (Malvern Instruments, Worcestershire, UK) at pH 2–9 in 10 mM ionic strength phosphate-buffered saline (PBS) as described in detail previously by Kurniawan and Fukuda [4].

### 2.5. Adsorption Kinetics

The biofilm pellets were divided into 2 parts. The first part was washed six times with 5 mM PBS at pH 7 by centrifugation. This biofilm was used to examine the kinetics of NH_4_^+^ adsorption. The second part of biofilm was washed six times with distilled water. This biofilm was used to examine the kinetics of NO_3_^−^ adsorption. The distilled water was used instead of PBS to avoid the influence of the anion in the PBS on NO_3_^−^ adsorption to the biofilms. All the biofilm pellets were stored at −40°C prior to ion adsorption analysis.

One wet-g of the biofilm pellet was resuspended in 50 mL of 5 mM PBS at pH 7. The suspension was mixed vigorously with a vortex for 5 min and then sonicated for 10 min, followed by vortexing for 30 s. Then, 5.0 mL of a 20 mM solution of reagent grade NH_4_Cl or NaNO_3_ prepared by diluting the chemical compound (Wako Pure Chemical Industries, Osaka, Japan) in 5 mM PBS at pH 7 was added to the suspension. The temperature of the suspension was maintained in an ice bath (approximately 0°C) and mixed well using a magnetic stirrer. The aliquots of the suspension were subsampled after various intervals (0.5, 1, 3, 5, 10, 20, 30, and 60 minutes) and then centrifuged (15,000 ×g at 4°C for 1 min) to separate the supernatant and the pellet. The ion concentration in the solution was measured using a capillary electrophoresis method (CAPI-3300, Otsuka electronics, Osaka, Japan). Fifty milliliters of the 5 mM phosphate buffer (pH 7) was used as the control for the experiments. The quantity of ions adsorbed to the biofilm was calculated from the difference between the ion concentrations in the subsamples and the control.

## 3. Results and Discussion

### 3.1. Nutrient Ions inside and outside the Biofilm

**Figure 1:**
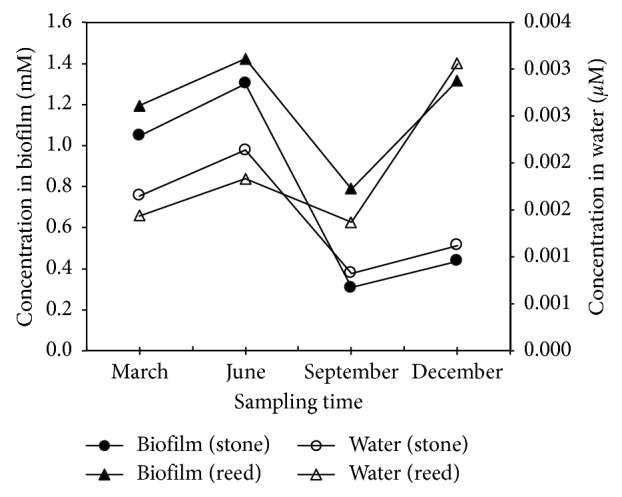
NH_4_^+^ concentrations inside the biofilm (formed on stones and reeds) and in the surrounding waters. See the left axis for the biofilm and the right axis for the lake water. Solid symbols (• for stone and ▲ for reed) and open symbols (○ for stone and △ for reed) indicate the ion concentrations in the biofilms and the surrounding lake water, respectively.

**Figure 2:**
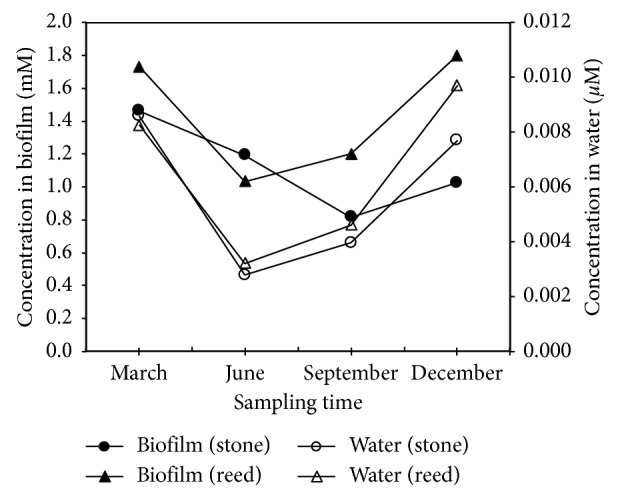
NO_3_^−^ concentrations inside the biofilm (formed on stones and reeds) and in the surrounding waters. See the left axis for the biofilm and the right axis for the lake water. Solid symbols (• for stone and ▲ for reed) and open symbols (○ for stone and △ for reed) indicate the ion concentrations in the biofilms and the surrounding lake water, respectively.

The nutrient ion concentrations (*i.e*., NH_4_^+^ and NO_3_^−^) in the interstitial water of the biofilm matrices were investigated for approximately one year with 3-month sampling intervals (4 sampling time points). The results were compared to the concentrations of the ions in the water surrounding the biofilm matrices. The concentrations of both NH_4_^+^ (Figure 1) and NO_3_^−^ (Figure 2) were much higher (hundreds to thousands of times) than the concentrations in the surrounding lake water. These results indicate that the microenvironment inside the biofilm is a nutrient-rich microhabitat.

The concentrations of nutrient ions inside the biofilm matrices dynamically change in synchrony with the changes in the ion concentrations in the lake water. This result suggested that the ion concentrations inside the biofilm were closely connected to the ion concentrations in the surrounding lake water. Related to these findings, our previous results showed that the internal regions of the biofilms might dynamically attract nutrient ions from the outside environment [12]. It seems that when the concentration of ions in surrounding water of biofilm matrices increases and becomes higher than the previous equilibrium state of ions between the biofilm and surrounding water, the biofilm seems to be able to accumulate ions from surrounding water through an attractive electrostatic interaction and ion-exchange mechanism until a new equilibrium state of ions is achieved. On the contrary, when the ion concentrations in surrounding water of the biofilm matrices decrease and become lower than the previous equilibrium state, the biofilm will release ions to the surrounding environment till a new equilibrium state of ions between the biofilm and surrounding water is attained. These suggest that the internal regions of biofilms were able to dynamically adapt to and exchange ions with the outside environment. This ability may lead to utilization of biofilms to stabilize the ion concentrations in aquatic environments.

The seasonal dynamic of nutrient ions in the lake water can be due to the influence of the environmental conditions [7]. Increases and decreases in water temperature and light intensity may affect the activity of photosynthesis resulting in the change of the nutrient ion concentration in the lake water [13]. The dynamic equilibrium between consumption and production of the ions may also affect the seasonal dynamic of nutrient ions in the lake water [14, 15]. However, further study to reveal the reason of the seasonal dynamic of the nutrient ions seems to be necessary.

### 3.2. Bacterial Community Structure

**Figure 3:**
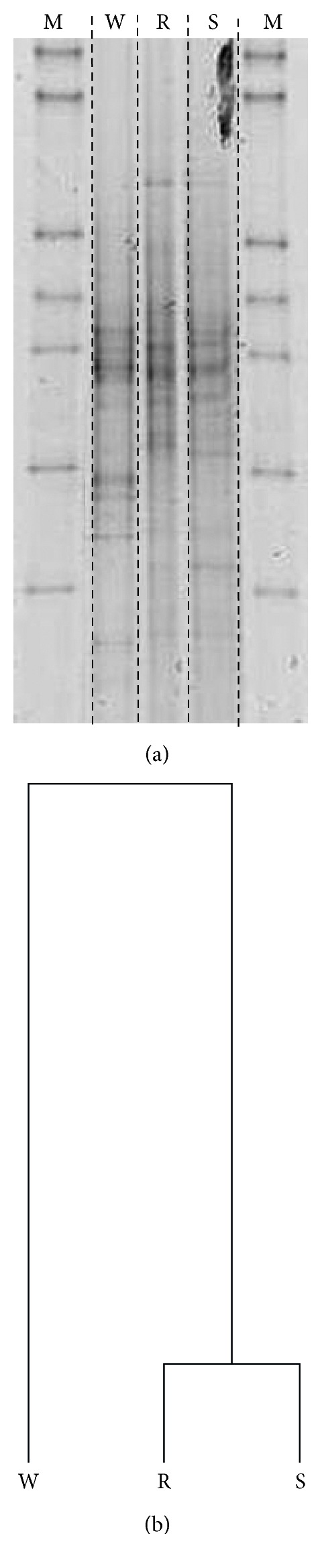
(a) Bacterial DGGE patterns of the amplified 16S rRNA genes from the biofilms on the stones (S) and reeds (R) and the surrounding water (W); (b) cluster analysis of the DGGE patterns of the amplified 16S rRNA genes from the biofilms on the stones (S) and reeds (R) and the surrounding water (W).

The nutrient-rich microenvironments inside the biofilm provide nutrients for microbes. Hence, the community structure of microbes inside the biofilm should be different from the community structure in the surrounding lake water due to the abundance of nutrients inside the biofilm. To evaluate this supposition, the microbial community structure inside the biofilm collected in December (the last sampling time point) was investigated and compared to that in the surrounding lake water (Figure 3).

The bacterial community structures differed between the biofilm matrices (formed on stone and reeds) and the lake water, as shown in the PCR-DGGE patterns and phylogenetic tree. The community structures inside the biofilms (stones and reeds) showed more similarity to one another than to the community structures in the surrounding lake water. The specific microhabitats inside the biofilm seem to affect microbial growth, resulting in a different community structure inside the biofilm than in the surrounding water [2, 12]. The number of bacteria in the biofilm is far greater (in the order of 10^9^ cells/wet-g) than in the lake water (in order of 10^6^ cells/wet-g). The nutrient-rich microhabitat inside biofilms (Figure 1) seems to have enhanced microbial growth resulting in the dense population of microbes inside the biofilm. The nutrient ions held inside the biofilm can be used by microbes and transformed into a biomass inside the biofilm. Hence, the biofilms may continuously grow and thus take more nutrient ions from the surrounding water.

### 3.3. Electrical Charge Properties

**Figure 4:**
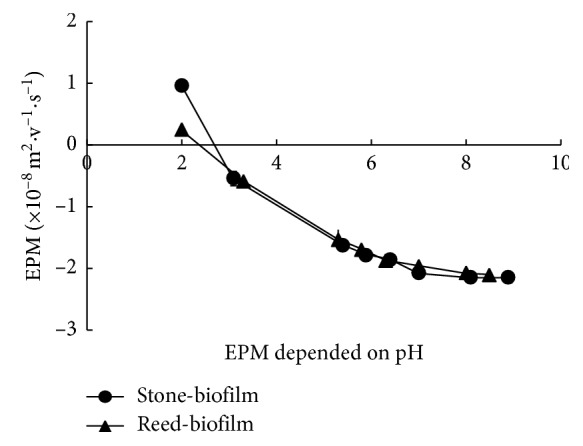
Electrophoretic mobility (EPM) of the biofilm (formed on stones and reeds) as a function of the pH. The EPM was measured under various pH conditions at a 10 mM ionic strength.

The accumulation of nutrient ions inside the biofilm has been reported to occur through electrostatic interactions (between the nutrient ions and charged sites of the biofilm polymers) and an ion-exchange mechanism [12]. The accumulated nutrient ions may be reserved on the charged sites of the biofilm polymers and the regions between the biofilm polymers. One of the main characteristics of the biofilm interiors that support this accumulation process is the electric charge properties of the biofilm polymer [16]. These properties were investigated for the biofilms in this study (Figure 4).

The EPM values of the biofilms formed on both the stones and reeds showed positive and negative charges. The negative EPM value at a pH higher than 5 and the significant shift in the EPM value at approximately pH 4 indicated the presence of functional groups with a negative charge, such as carboxylic groups, whereas the positive EPM value at approximately pH 2 revealed the existence of functional groups carrying a positive charge, such as amino groups [17]. The decrease of the negative charge in the biofilm polymers along with the decrease of the pH values seems due to the protonation of the functional group carrying negatively charged sites. The positive value of the EPM at pH 2 suggested that the positively charged sites can be detected after the negatively charged sites can be neutralized through protonation.

The results of the EPM measurement indicate that the biofilm carries both positively and negatively charged sites in aquatic ecosystems, which enable the biofilm to attract and accumulate both anionic and cationic nutrients, respectively. The negative charges measured around pH 7 indicate that the biofilm has a net negative charge in this pH. The net negative charge of the biofilms occurs due to the greater number of negatively charged sites than positively charged sites on the biofilm polymers [4, 18]. The charged sites of the biofilm play essential roles in attracting and conserving ions from the surrounding environments.

### 3.4. Enrichment of the Microhabitat inside the Biofilm

The adsorption of nutrient ions into the biofilm is thought to lead to the enrichment of nutrients inside the biofilm [8, 19, 20, 21]. To clarify this mechanism in more detail, the adsorption of nutrient ions (*i.e*., NH_4_^+^ and NO_3_^−^) to the biofilm was investigated in this study. The sample used was the biofilm formed on stones collected in December. In this case, the biofilms formed on reeds could not be used due to the limitations of these biofilm samples. The main focuses of the investigation were the adsorption kinetics and the adsorption isotherms of the nutrient ions.

**Figure 5:**
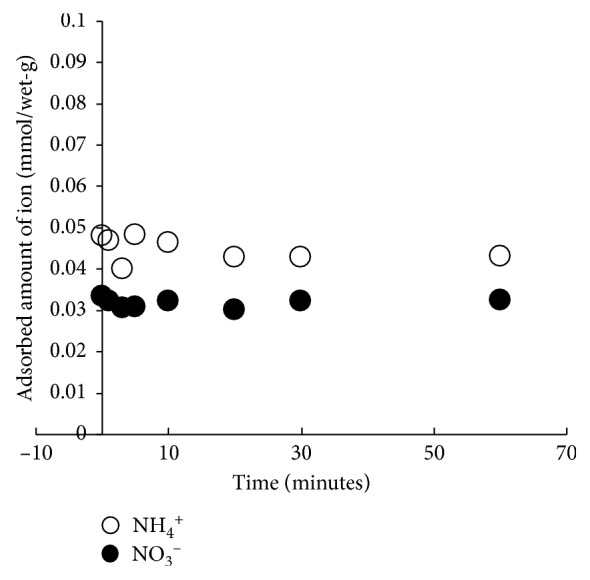
Time course of nutrient ion accumulation in the biofilm formed on the stones. NH_4_^+^ and NO_3_^−^ are indicated by open (○) and solid (•) symbols, respectively.

The time course of nutrient ion adsorption to the biofilm was investigated (Figure 5). All nutrient ions examined (*i.e*., NH_4_^+^ and NO_3_^−^) were adsorbed to the biofilm in a short time span. The adsorption amount attained within 1 min was not exceeded for the rest of the experiment. The fast adsorption process (*i.e*., within 5 minutes) is typical of the adsorption that occurs due to a physicochemical process. Hence, adsorption of NH_4_^+^ and NO_3_^−^ on the biofilm seems to occur as the physicochemical process, with the electrostatic forces between the ions and the negatively charged sites in the biofilm polymer serving as the driving force [12]. There is the possibility that the adsorption of ions may occur more after longer contact times such as after several days due to other mechanisms such as active uptake accumulation promoting microbial metabolisms [22].

The enrichment of nutrient ions inside the biofilm suggests the removal of these ions from outside the biofilm [14, 23–28]. The nutrient ions held in the biofilm can be easily used by microbes and transformed into a biomass inside the biofilm; thus, the ions may be continuously attracted from the surrounding lake water [25, 29, 30]. These characteristics of the biofilm may contribute to the suppression of excess nutrient ions outside the biofilm, such as in lakes, rivers, or ponds [31, 32].

The present studies investigated the characteristics of the microenvironment inside biofilm of natural microbial consortia to analyze the influence of the nutrient ion accumulation inside the biofilm to the seasonal dynamic of the ions in aquatic ecosystems. The results show the following: (1) the interior inside the biofilm is nutrient rich and changes in synchrony with the surrounding water; (2) the bacterial community structure differs between the biofilm and the surrounding water; (3) biofilm polymers have both positive and negative charges; (4) the attractive electrostatic interactions between the charges on the biofilm polymers and the oppositely charged ions outside the biofilm seem to significantly influence the enrichment of nutrient ions inside the biofilm matrices. The enrichment of ions inside the biofilm suggested the removal of these ions from the water outside the biofilm. Microbes can utilize the nutrient ions that are held between the biofilm polymers and transformed into biomass inside the biofilm. Hence, the biofilm may continuously accumulate the ions from surrounding water. This function of the biofilm may lead to suppression of pollution or excess nutrient ions outside the biofilm.

## Data Availability

The data used to support the findings of this study are included in the article.
